# Linear association of the dietary index for gut microbiota with insulin resistance and type 2 diabetes mellitus in U.S. adults: the mediating role of body mass index and inflammatory markers

**DOI:** 10.3389/fnut.2025.1557280

**Published:** 2025-03-21

**Authors:** Haoran Qu, Yiyun Yang, Qihang Xie, Liu Ye, Yue Shao

**Affiliations:** ^1^Department of Cardiothoracic Surgery, The First Affiliated Hospital of Chongqing Medical University, Chongqing, China; ^2^Department of Anesthesiology, Children's Hospital of Chongqing Medical University, Chongqing, China; ^3^Department of Health Management Center, The First Affiliated Hospital of Chongqing Medical University, Chongqing, China

**Keywords:** gut microbiota, type 2 diabetes mellitus, insulin resistance, BMI, inflammatory marker, NHANES

## Abstract

**Background:**

Gut microbiota is reported to be related to the onset of insulin resistance (IR) and type 2 diabetes mellitus (T2DM). The dietary index for gut microbiota (DI-GM) is a novel index for reflecting gut microbiota diversity. We aimed to evaluate the association of DI-GM with T2DM and IR.

**Methods:**

This cross-sectional research comprised 10,600 participants aged ≥20 from the National Health and Nutrition Examination Survey (NHANES) 2007–2018. We employed weighted multivariable linear and logistic regression models to examine the correlation of DI-GM with T2DM and IR. Linear or nonlinear relationships were examined by restricted cubic spline (RCS) regression. Additionally, subgroup and sensitivity analyses were performed to ensure the reliability of the results. Mediation analysis explored the roles of body mass index (BMI) and inflammatory factors in these associations.

**Results:**

Higher DI-GM were inversely associated with T2DM (OR = 0.93, 95%CI: 0.89–0.98) and IR (OR = 0.95, 95%CI: 0.91–0.99) after adjusting for confounders. DI-GM ≥ 6 group showed significantly lower risks of T2DM (OR = 0.74, 95%CI: 0.60–0.91) and IR (OR = 0.77, 95%CI: 0.62–0.95). RCS demonstrated a linear relationship between DI-GM and T2DM, as well as IR. DI-GM was also inversely correlated with the risk markers of T2DM. Mediation analysis showed that BMI and the systemic inflammation response index partly mediated the association of DI-GM with T2DM and IR, while the systemic immune-inflammation index mediated only the association with T2DM.

**Conclusion:**

DI-GM is inversely associated with T2DM and IR, with BMI and inflammatory markers partly mediating this association.

## 1 Introduction

The high incidence of diabetes has become a critical health problem worldwide. The global incidence is estimated at 9.3% of the adult population in 2019, with projections indicating a significant increase (1). Type 2 diabetes mellitus (T2DM) represents the predominant form of diabetes, the burden of T2DM extends beyond its health implications, imposing significant economic challenges on healthcare systems worldwide (2). Diabetes care accounts for one-quarter of healthcare expenditures, with 61% directly attributable to the disease in the U.S. (3). Additionally, insulin resistance (IR) is a pathological condition marked by reduced cellular responsiveness to insulin. IR is typically accompanied by hyperinsulinemia and is closely linked to the onset of T2DM (4). Therefore, early detection and timely management of T2DM and IR are imperative.

In recent years, studies of the gut microbiota have already become a hotspot, and its association with T2DM has been widely discussed. Multiple studies suggested that the gut microbiota was associated with the occurrence and progression of T2DM and could represent a promising therapeutic target (5, 6). Furthermore, several studies suggested that modulating gut microbiota through dietary interventions is a promising approach to improving host health (7). The dietary index for gut microbiota (DI-GM) is a new tool developed by Kase et al. (8) for reflecting the diversity of gut microbiota. They identified 14 dietary components, having a positive or a negative effect on gut microbiota by systematically reviewing the literature and using these components to calculate the DI-GM. Additionally, they demonstrated that DI-GM was significantly related to urinary enterolignans, highlighting an association with gut microbiota diversity. This tool may help to design effective dietary patterns to prevent or alleviate dysbiosis-related diseases. Currently, a few studies have explored the relationship between DI-GM and other medical conditions (9, 10). There is a lack of studies discussing the association of DI-GM with T2DM and IR. Therefore, it’s necessary to explore if healthy gut microbiota dietary pattern identified by DI-GM was associated with a reduced risk of T2DM and IR.

Obesity is a major T2DM risk factor, visceral fat accumulation leads to adipokines and pro-inflammatory cytokines secretion, which contributes to systemic inflammation and IR (11, 12). Chronic low-grade inflammation is usually regarded to play a crucial role in the progression of IR and T2DM (13). Studies indicated that gut microbiota disorders leading to increased intestinal permeability can trigger low-grade systemic inflammation, this is a crucial risk factor in the onset of IR and obesity, leading to T2DM development (14). Therefore, we hypothesized that following a promoting healthy gut microbiota dietary pattern identified by DI-GM could mitigate T2DM and IR risk by reducing obesity and systemic inflammation.

This study aimed to investigate the correlation of DI-GM with T2DM and IR by utilizing data from the National Health and Nutrition Examination Survey (NHANES) and to investigate the mediation of BMI and inflammatory markers in this relationship. This study may offer new perspectives for the prevention and control of T2DM.

## 2 Materials and methods

### 2.1 Data sources and study population

All data obtained in this research is from the NHANES, which employs a cross-sectional, multistage, stratified, and subgroup probability sampling design to evaluate the nutritional and health conditions of the U.S. population.

**Figure 1:**
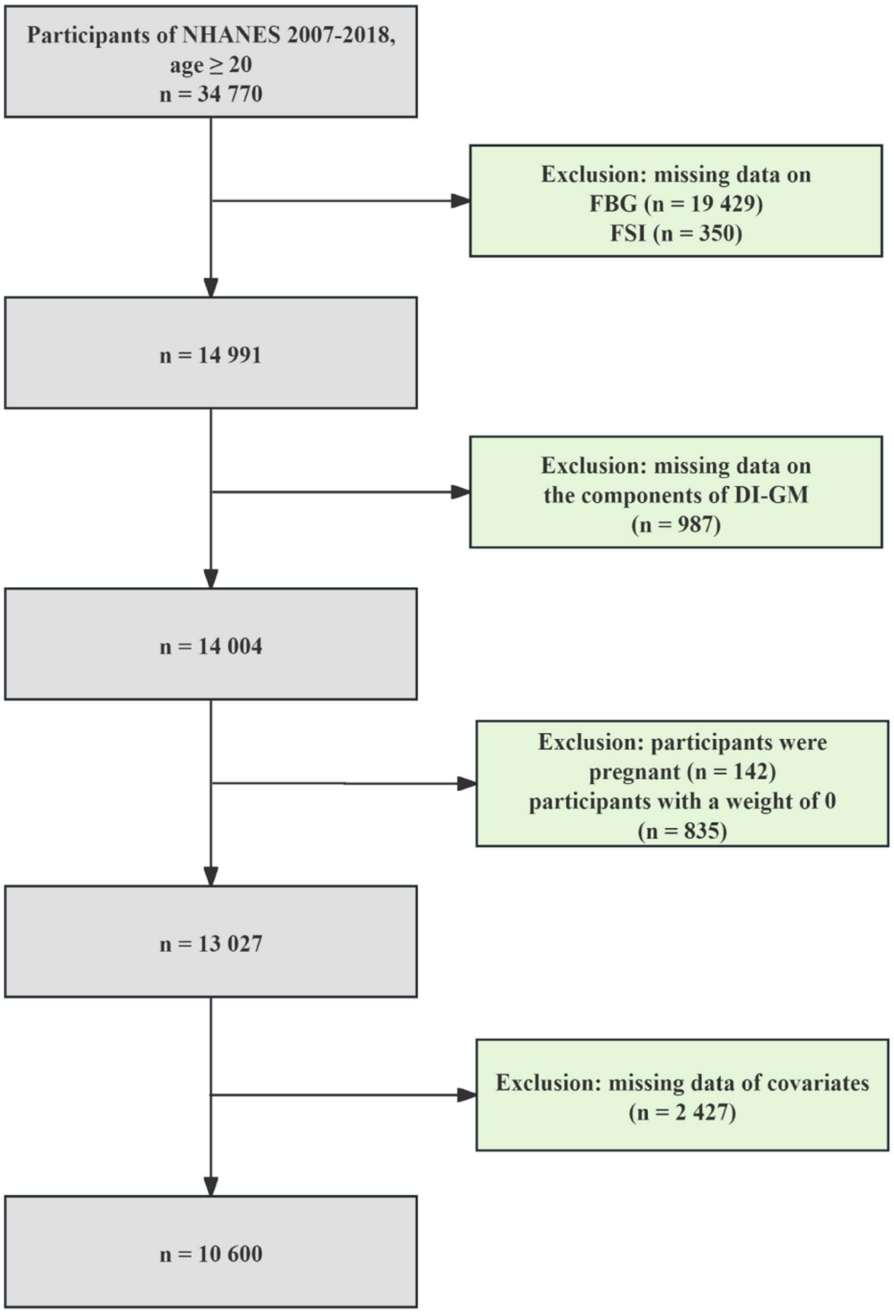
Participant screening flow chart.

We pooled six NHANES cycles data from 2007 to 2018 for this study, involving 34,770 participants aged ≥20 years. The exclusion of participants was determined based on the following criteria: (1) missing data on the diagnosis of T2DM (*n* = 19,429) and IR (*n* = 350); (2) missing information on DI-GM (*n* = 987); (3) participants were pregnant (*n* = 142); (4) participants with a weight of 0 (*n* = 835); (5) missing data on covariates (*n* = 2,427). Finally, 10,600 eligible participants were enrolled. The specific processes are shown in Figure 1.

### 2.2 Evaluation of the DI-GM

The dietary data are jointly gathered by the National Center for Health Statistics (NCHS) and the U.S. Department of Agriculture (USDA) through the What We Eat in America (WWEIA) survey (15). Trained surveyors utilize the computer-assisted USDA Automated Multiple-Pass Method to conduct irregular dietary recalls, asking participants about their consumption of beverages, foods, and dietary supplements from the previous day (16). An additional dietary recall was administered by telephone approximately 3–10 days following the initial assessment. An average of the values from both days was calculated for each diet component.

The DI-GM was constituted of 14 food items or nutrients, following the evaluation standards outlined in the article by Kase et al., including coffee, green tea, fermented dairy, avocado, broccoli, chickpeas, cranberries, fiber, whole grains, and soybean as beneficial elements, while unfavorable elements included processed meat, red meat, a high-fat diet (≥ 40% energy from fat), and refined grains (8). Participants whose consumption was exceeding the sex-specific median for beneficial elements or below the sex-specific median for unfavorable elements were assigned a score of 1. Conversely, a score of 0 was given for consuming below the sex-specific median for beneficial elements or exceeding the sex-specific median for unfavorable elements. DI-GM is obtained by summing the scores for each element, yielding a range from 0 to 14. Specific details are given in the Supplementary Table S1. Based on previous research, DI-GM scores were divided into three group: 0–3, 4–5, and ≥ 6 points (9).

### 2.3 Outcomes

T2DM, IR, and associated risk markers, including fasting blood glucose (FBG, mmol/L), fasting serum insulin (FSI, μU/ml), and homeostasis model assessment of insulin resistance (HOMA-IR) (17, 18) were considered as outcome variables.

In our study, T2DM was diagnosed as any of the following criteria: (1) participants with a self-reported diabetes diagnosis; (2) FBG level ≥ 7.0 mmol/L; (3) 2-h oral glucose tolerance test (OGTT) plasma glucose ≥11.1 mmol/L; (4) a glycosylated hemoglobin A1c (HbA1c) level ≥ 6.5% (19). Although the NHANES database does not provide explicit information on the specific types of diabetes, this study likely enrolled a majority of T2DM subjects since the proportion of type 1 diabetes mellitus (T1DM) in adulthood is low.

IR was evaluated by HOMA-IR (HOMA-IR = FSI (μU/ml) * FBG (mmol/L) / 22.5). Based on previous studies, the values greater than or equal to the 75th percentile were diagnosed as IR (HOMA-IR > 4.53 in our study) (20).

### 2.4 Definitions of mediating variables

The determination of the anthropometric, complete blood count test and basic biochemical characteristics of the participants in NHANES have been reported in previous publications (21–24). Inflammatory markers included the Systemic Immune Inflammation Index (SII), the Systemic Inflammation Response Index (SIRI). SII and SIRI were widely used in research to quantify systemic inflammation, calculated by neutrophil count (NEUT), platelet count (PLA), lymphocyte count (LYM), and monocyte count (MONO). The calculation formulas are as follows: SII = (NEUT × PLA) / LYM, SIRI = (NEUT × MONO) / LYM (24–26).

BMI was calculated through the standard formula: BMI = weight/height^2^ (27).

### 2.5 Covariates

Covariates were selected based on some published studies (9, 17, 28). Therefore, we included the following covariables: age, race, gender, education level, poverty-to-income ratio (PIR), marital status, smoking status, drinking status, physical activity (PA), BMI, whether they have hypertension, hyperlipidemia, hyperuricemia and cardiovascular disease (CVD). The definition and measurement of the covariates are shown in the Supplementary Appendix.

### 2.6 Statistical analysis

All analyses considered the fasting subsample weights (WTSAF2YR) and were divided by six to obtain pooled weights across six survey cycles, as this constituted the smallest subsample of the study, according to NHANES analysis guidelines.

Continuous variables are presented as mean ± standard deviation (SD) or median (interquartile range, IQR), and other variables are reported as weighted percentages (%). Differences between participants grouped by DI-GM scores (0–3, 4–5, ≥6) were analyzed using a weighted t-test (for continuous variables) and a weighted chi-square test (for categorical variables). Pairwise comparisons (eg, 0–3 vs 4–5, 0–3 vs ≥ 6, and 4–5 vs ≥ 6) were considered to be statistically significant if the corresponding unadjusted pairwise *p* ≤ 0.017 (ie, < 0.05/3 for Bonferroni correction).

Multivariable weighted logistic and linear regression models were utilized to investigate the association of DI-GM with T2DM, IR, and the risk markers of T2DM (FBG, FSI, and HOMA-IR). The Odds ratios (ORs), Beta (*β*) values, and 95% confidence intervals were calculated, respectively. DI-GM was analyzed as a continuous variable and a grouped variable (0–3, 4–5, ≥6). Model 1 was unadjusted. Model 2 was adjusted by age, gender, and race. Model 3 was adjusted for PIR, marital status, education level, BMI, smoking status, drinking status, PA, hypertension, hyperlipidemia, hyperuricemia, and CVD based on Model 2.

Linear or non-linear associations of DI-GM with T2DM and IR were investigated by restricted cubic splines (RCS), adjusting for all confounding variables in model 3. Additionally, we performed interaction and subgroup analyses to investigate potential confounders affecting the association of DI-GM with T2DM and IR. Subgroup analyses were detected by stratifying by sex, age, race, BMI, PA, hypertension, hyperuricemia, hyperlipidemia, and CVD. Mediation analyses (1,000 bootstraps) were conducted to assess the mediation effect of BMI and inflammatory markers on the association of DI-GM with T2DM and IR. To explore the potential inflammatory pathways, we also conducted mediation analyses on white blood cells (WBC) and the inflammatory cells included in the two indicators (NEUT, LYM, and MONO).

Sensitivity analyses were conducted to evaluate the reliability of the results. First, we applied multiple imputation (MI) by chained equations to input the missing data on covariates (9, 28). Subsequently, the imputed dataset was transferred to weighted multifactor regression analysis. Second, unweighted regression analyses were conducted. Third, further adjustments were made for liver and kidney function (alanine aminotransferase, aspartate aminotransferase, *γ*-glutamyl transpeptidase, alkaline phosphatase, serum creatinine, blood urea nitrogen) and total energy intake (kcal/day) to evaluate whether these factors influenced the association of DI-GM with T2DM and IR. Finally, given the close association between CVD, hyperuricemia and T2DM, we performed regression analyses again after excluding participants who had CVD or hyperuricemia at baseline.

All statistical analyses were completed with R software version 4.2.2 and Free Statistics software version 2.0. A two-sided *p* < 0 0.05 was considered as statistically significant.

## 3 Results

### 3.1 Participants characteristics

The baseline characteristics of participants stratified by the DI-GM scores are shown in Table 1. The mean age of the participants was 47.10 ± 16.71 years. The proportion of participants diagnosed with T2DM was 15.01%, while 21.22% were defined as having IR. Compared with the group with the lowest DI-GM level, participants in the highest DI-GM group were predominantly older, predominantly female, Non-Hispanic White, and more likely to be married or living with a partner. Additionally, this group exhibited higher levels of physical activity, education, and PIR. They also had a lower BMI, were less likely to smoke or engage in heavy drinking, and had a lower prevalence of T2DM and IR.

**Table 1:** Weighted characteristics of participants grouped by the DI-GM values.

Variables	Overall	0–3	4–5	≥6	*p*-value
*n*	10,600	2,602	5,036	2,962	
Age (years), mean ± SD	47.10 ± 16.71	45.31 ± 16.59^b^	46.35 ± 16.75 ^c^	49.66 ± 16.47	<0.001
Gender (%)					0.006
Male	50.00	53.20^b^	50.06	47.44	
Female	50.00	46.80	49.94	52.56	
Race (%)					<0.001
Non-Hispanic Black	10.36	14.19^a, b^	10.83^c^	6.65	
Non-Hispanic White	69.38	65.74	66.93	76.00	
Mexican American	8.07	8.55	9.33	5.74	
other	12.20	11.52	12.91	11.61	
Education level (%)					<0.001
Less than High school	14.77	17.99^a, b^	16.09^c^	10.21	
High school grad or equivalent	22.68	29.48	22.00	18.50	
College or above	62.55	52.53	61.91	71.29	
Marital status (%)					0.001
Married or living with partner	64.18	62.04^b^	63.04^c^	67.62	
Other	35.82	37.96	36.96	32.38	
PIR (%)					<0.001
<1.3	20.76	24.54^a, b^	23.09^c^	14.21	
1.3–3.5	36.18	40.94	35.27	33.91	
>3.5	43.06	34.53	41.64	51.88	
BMI (kg/m2) (%)					<0.001
<25	29.71	24.65^a, b^	29.05^c^	34.63	
≥25	70.29	75.35	70.95	65.37	
Smoking status (%)					<0.001
Never	55.51	54.79^b^	55.23^c^	56.49	
Former	25.46	23.58	23.97	29.25	
Current	19.03	21.63	20.80	14.26	
Drinking status (%)					<0.001
Never	10.13	10.20^b^	10.94^c^	8.79	
Former	38.44	34.01	36.29	45.21	
Mild	17.71	17.82	17.45	18.04	
Moderate	21.37	24.76	22.60	16.85	
Heavy	12.35	13.22	12.72	11.11	
PA (%)					<0.001
No	24.98	27.25^b^	26.18^c^	21.34	
Yes	75.02	72.75	73.82	78.66	
Hypertension (%)					0.727
No	62.34	61.44	62.59	62.64	
Yes	37.66	38.56	37.41	37.36	
Hyperlipidemia (%)					0.792
No	29.47	28.96	29.35	30.04	
Yes	70.53	71.04	70.65	69.96	
Hyperuricemia (%)					0.402
No	79.32	78.82	78.89	80.38	
Yes	20.68	21.18	21.11	19.62	
CVD (%)					0.460
No	91.29	91.13	90.98	91.92	
Yes	8.71	8.87	9.02	8.08	
T2DM (%)					0.004
No	84.99	82.81^b^	84.95	86.74	
Yes	15.01	17.19	15.05	13.26	
IR (%)					<0.001
No	78.78	75.72^b^	77.56^c^	83.06	
Yes	21.22	24.28	22.44	16.94	
FBG (mmol/L), mean ± SD	5.91 ± 1.65	6.06 ± 1.94^a, b^	5.89 ± 1.61	5.83 ± 1.47	<0.001
HbA1c (%), mean ± SD	5.62 ± 0.92	5.69 ± 1.06^a, b^	5.62 ± 0.90	5.57 ± 0.82	<0.001
FSI (μU/ml), median [IQR]	9.45 [6.00, 15.38]	10.16 [6.46, 16.85] ^b^	9.63 [6.12, 15.77] ^c^	8.55 [5.48, 13.67]	<0.001
HOMA-IR, median [IQR]	2.39 [1.43, 4.12]	2.65 [1.58, 4.43]^a, b^	2.45 [1.46, 4.23] ^c^	2.12 [1.33, 3.68]	<0.001

Mean ± SD and Median (IQR) for continuous variables, *p*-value was calculated by weighted t-test. % for categorical variables, *p*-value was calculated by weighted chi-square test.

^a^0–3 vs 4–5 group, the significance level is based on Bonferroni-adjusted p < 0.017.

^b^0–3 vs ≥ 6 group, the significance level is based on Bonferroni-adjusted p < 0.017.

^c^4–5 vs ≥ 6 group, the significance level is based on Bonferroni-adjusted p < 0.017.

DI-GM, dietary index for gut microbiota; PIR, Poverty Income Ratio; PA, Physical Activity; CVD, Cardiovascular Disease; T2DM, type 2 diabetes mellitus; IR, insulin resistance; FBG, fasting blood glucose; HbA1c, glycosylated hemoglobin A1c; FSI, fasting serum insulin; HOMA-IR, homeostasis model assessment of insulin resistance; SD, Standard Deviation; IQR, Interquartile Range.

### 3.2 Associations of DI-GM with T2DM and IR

Table 2 illustrates the associations of DI-GM with T2DM and IR. The results of analysis when including DI-GM as a continuous variable have shown that DI-GM is inversely correlated with T2DM occurrence (OR = 0.93, 95% CI = 0.90, 0.97), the association remained robust after adjustment (OR = 0.93, 95% CI = 0.89, 0.98), and so was the association of DI-GM with IR (OR = 0.95, 95% CI = 0.91, 0.99). Moreover, after grouping DI-GM, DI-GM ≥ 6 group participants were inversely associated with the risk of T2DM, compared to those in the lowest DI-GM group in model 3 (OR = 0.74, 95% CI = 0.60, 0.91), and the trend test was significant (*P* for trend = 0.006). Meanwhile, the relationship between DI-GM and IR was also significantly negative (OR = 0.77, 95% CI = 0.62, 0.95) with the *P* for trend = 0.014 in model 3.

**Table 2:** The associations of DI-GM with T2DM and IR.

Outcomes	Model 1 OR (95%CI)	*p-*value	Model 2 OR (95%CI)	*p-*value	Model 3 OR (95%CI)	*p-*value
T2DM						
DI-GM continuous	0.93 (0.90, 0.97)	<0.001	0.89 (0.85, 0.93)	<0.001	0.93 (0.89, 0.98)	0.005
DI-GM group						
0–3	Reference		Reference		Reference	
4–5	0.85 (0.74, 0.98)	0.030	0.80 (0.68, 0.93)	0.004	0.85 (0.73, 1.00)	0.047
≥6	0.74 (0.62, 0.87)	<0.001	0.60 (0.50, 0.73)	<0.001	0.74 (0.60, 0.91)	0.006
*P* for trend		<0.001		<0.001		0.006
IR						
DI-GM continuous	0.91 (0.87, 0.94)	<0.001	0.90 (0.87, 0.94)	<0.001	0.95 (0.91, 0.99)	0.030
DI-GM group						
0–3	Reference		Reference		Reference	
4–5	0.90 (0.77, 1.06)	0.199	0.89 (0.76, 1.05)	0.161	0.98 (0.82, 1.17)	0.798
≥6	0.64 (0.53, 0.77)	<0.001	0.62 (0.51, 0.75)	<0.001	0.77 (0.62, 0.95)	0.016
*P* for trend		<0.001		<0.001		0.014

Model 1: adjusted for no covariates; Model 2: adjusted for age, gender, race; Model 3: adjusted for all covariates. (age, gender, race, education level, marital status, PIR, smoking status, drinking status, PA, BMI, hypertension, hyperlipidemia, hyperuricemia and CVD).

OR, odds ratio; 95% CI, 95% confidence interval.

**Figure 2:**
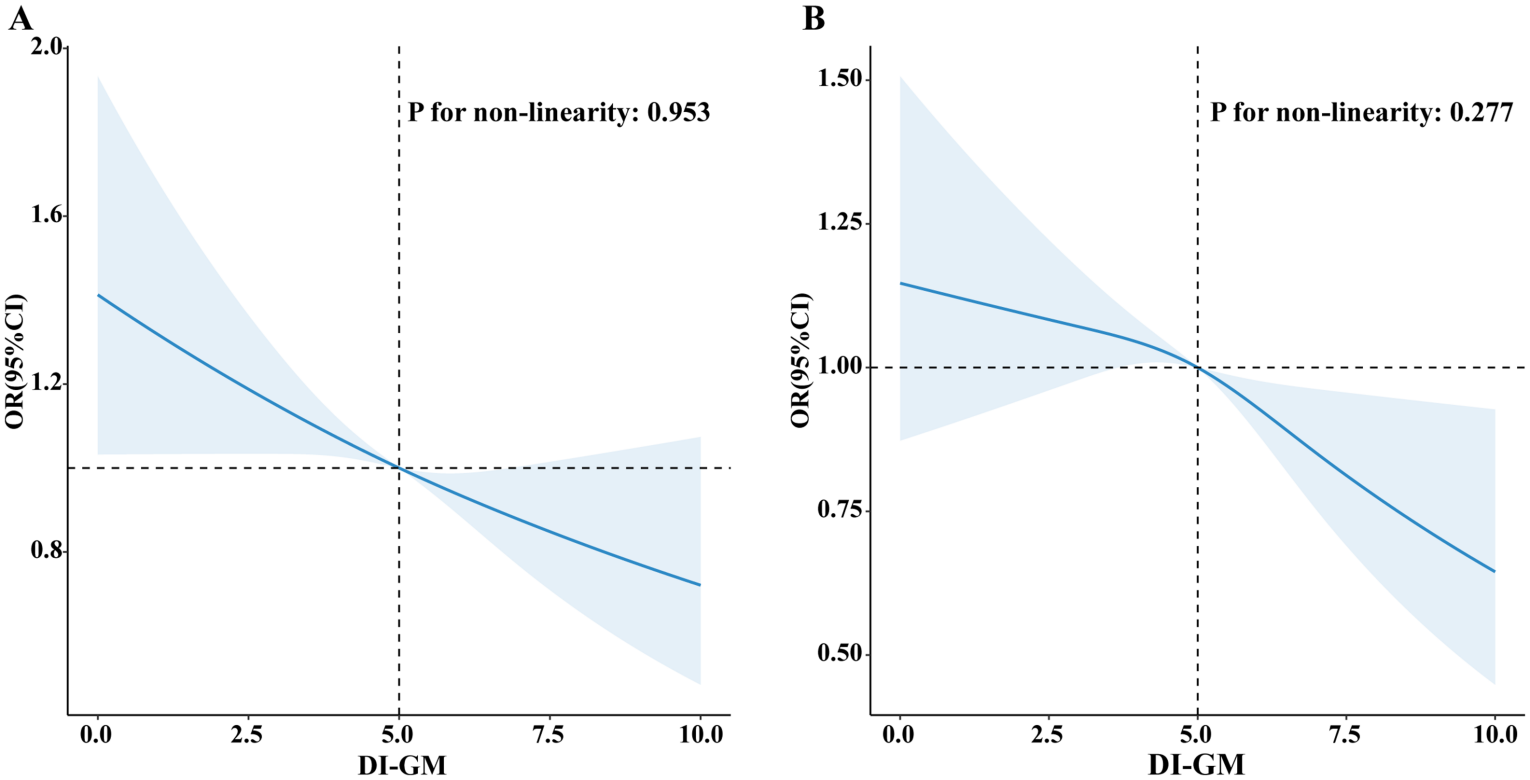
Association of DI-GM with T2DM and IR by RCS. **(A)** T2DM; **(B)** IR.

Figure 2 illustrates a visual representation of the relationship between DI-GM and T2DM as well as IR. The RCS revealed a linear association between DI-GM and T2DM (non-linearity: *p* = 0.953), as well as IR (non-linearity: *p* = 0.277) in the fully adjusted models.

### 3.3 Relationship between DI-GM and risk markers of T2DM

Table 3 presents the relationship between DI-GM and risk markers for T2DM. The DI-GM was inversely associated with FBG (*β* = −0.03, 95% CI = −0.05, 0.00), FSI (*β* = −0.19, 95% CI = −0.34, −0.04), and HOMA-IR (*β* = −0.10, 95% CI = −0.16, −0.04) as a continuous variable after adjusting for all confounders. Moreover, when used as a categorical variable, the lowest DI-GM group was used as a reference, the associations of DI-GM ≥ 6 with FBG (*β* = −0.16, 95% CI = −0.27, −0.06), FSI (*β* = −0.86, 95% CI = −1.66, −0.06), and HOMA-IR (*β* = −0.48, 95% CI = −0.81, −0.15) were also significant negative, with all *P* for trend <0.05 in the full adjusted model.

**Table 3:** The association between DI-GM and risk markers of T2DM.

Outcomes	Model 1 *β* (95% CI)	*p*-value	Model 2 *β* (95% CI)	*p*-value	Model 3 *β* (95% CI)	*p*-value
FBG						
DI-GM continuous	−0.04 (−0.07, −0.02)	<0.001	−0.06 (−0.08, −0.03)	<0.001	−0.03 (−0.05, 0.00)	0.028
DI-GM group						
0–3	Reference		Reference		Reference	
4–5	−0.16 (−0.26, −0.07)	<0.001	−0.18 (−0.27, −0.09)	<0.001	−0.13 (−0.22, −0.05)	0.003
≥6	−0.23 (−0.33, −0.12)	<0.001	−0.28 (−0.38, −0.18)	<0.001	−0.16 (−0.27, −0.06)	0.002
*P* for trend		<0.001		<0.001		0.003
FSI						
DI-GM continuous	−0.55 (−0.71, −0.39)	<0.001	−0.53 (−0.68, −0.37)	<0.001	−0.19 (−0.34, −0.04)	0.012
DI-GM group						
0–3	Reference		Reference		Reference	
4–5	−0.45 (−1.40, 0.50)	0.346	−0.46 (−1.41, 0.49)	0.341	0.03 (−0.84, 0.89)	0.951
≥6	−2.30 (−3.13, −1.47)	<0.001	−2.22 (−3.04, −1.39)	<0.001	−0.86 (−1.66, −0.06)	0.035
*P* for trend		<0.001		<0.001		0.022
HOMA-IR						
DI-GM continuous	−0.21 (−0.27, −0.15)	<0.001	−0.21 (−0.27, −0.15)	<0.001	−0.10 (−0.16, −0.04)	0.002
DI-GM group						
0–3	Reference		Reference		Reference	
4–5	−0.30 (−0.69, 0.09)	0.127	−0.31 (−0.70, 0.07)	0.110	−0.15 (−0.51, 0.22)	0.421
≥6	−0.92 (−1.24, −0.59)	<0.001	−0.94 (−1.27, −0.61)	<0.001	−0.48 (−0.81, −0.15)	0.006
*P* for trend		<0.001		<0.001		0.003

Model 1: adjusted for no covariates; Model 2: adjusted for age, gender, race; Model 3: adjusted for all covariates. (age, gender, race, education level, marital status, PIR, smoking status, drinking status, PA, BMI, hypertension, hyperlipidemia, hyperuricemia and CVD).

FBG, fasting blood glucose; FINS, fasting serum insulin; HOMA-IR, homeostasis model assessment of insulin resistance.

### 3.4 Subgroup analyses

**Figure 3:**
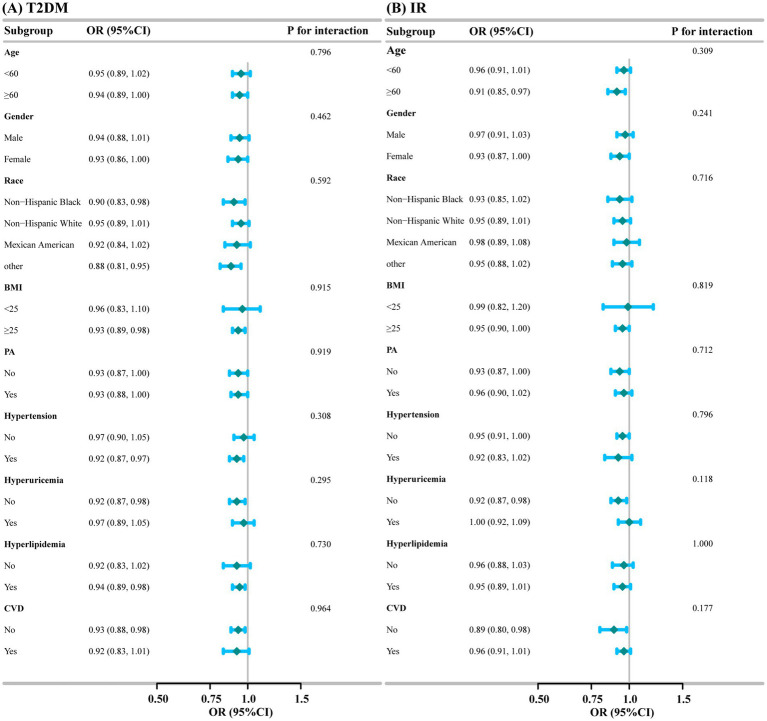
Subgroup analysis of the association of DI-GM with T2DM and IR. **(A)** T2DM; **(B)** IR.

Subgroup and interaction analyses were conducted for the association of DI-GM with T2DM and IR based on age, gender, race, BMI, PA, Hypertension, Hyperuricemia, Hyperlipidemia, and CVD. As shown in Figure 3, subgroup analyses illustrated the stability of the results. We saw no significant interaction between DI-GM and T2DM (All *P* for interaction>0.05), nor between DI-GM and IR (All *P* for interaction >0.05).

### 3.5 Mediation analyses

**Figure 4:**
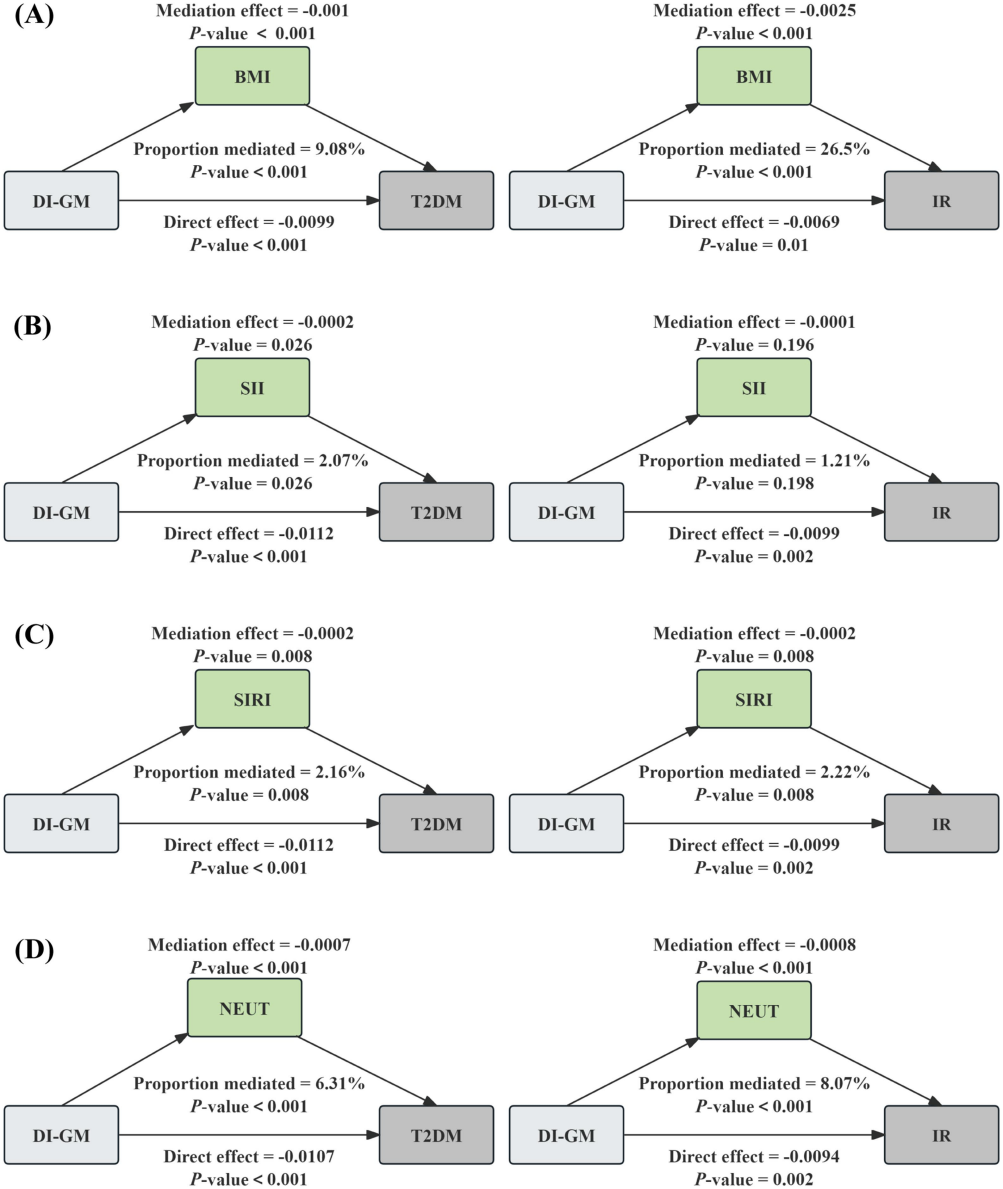
The mediation analysis of BMI, inflammatory biomarkers, and NEUT on the association of DI-GM with T2DM and IR. The graphs in **(A–D)** represented the mediating role of BMI, SII, SIRI, and NEUT, respectively.

We conducted the mediation analyses to explore the mediating effect of BMI and inflammatory markers. As shown in Figure 4, BMI significantly mediated the associations of DI-GM with T2DM and IR, explaining 9.08%, and 26.5%, respectively (*p* < 0.001). Meanwhile, two inflammatory markers had a significant mediation on the association between DI-GM and T2DM, SII and SIRI explained 2.07% (*p* = 0.026), and 2.16% (*p* = 0.008), respectively. In addition, SIRI partially mediated the relationship between DI-GM and IR, with a mediation ratio of 2.22% (*p* = 0.008), and SII had no significant mediating effect (*p* > 0.05). Further analysis revealed that NEUT mediated 6.31% (*p* < 0.001) of the association between DI-GM and T2DM and 8.07% (*p* < 0.001) of the association with IR. No significant mediation effects were observed for other inflammatory cells (all *p* > 0.05) as shown in Supplementary Figure S1.

### 3.6 Sensitivity analyses

In addition, sensitivity analyses were implemented. Firstly, we used multiple imputation for missing data on covariates, multivariable regression analyses were conducted subsequently. The results were shown in Supplementary Tables S2, S3, a significant inverse correlation between DI-GM and T2DM, IR, and risk markers of T2DM was found, which was consistent with the result of the main analysis. Secondly, the results of the unweighted multivariate regression analyses were similar to the weighted analyses, this further illustrates the robustness of the results, as shown in Supplementary Tables S4, S5. Thirdly, when further adjusting for the liver and kidney function indicators and total energy intake, respectively, the results remained unchanged significantly (Supplementary Tables S6, S7). Lastly, the results remained robust in participants without CVD (Supplementary Tables S8, S9) or hyperuricemia (Supplementary Tables S10, S11).

## 4 Discussion

The results of our study, which included 10,600 participants, demonstrated that DI-GM (continuous variable) and DI-GM ≥ 6 group were significantly inversely correlated with T2DM, IR, and the risk markers of T2DM (FBG, FSI, and HOMA-IR), the correlation remained significant after adjusting the covariates. RCS visualized a linear correlation of DI-GM with T2DM and IR. Moreover, subgroup and sensitivity analyses were conducted to further validate the reliability of the primary results. Mediation analysis showed that BMI and two inflammatory markers (SII and SIRI) partly mediated the associations of DI-GM with T2DM and IR.

The association between gut microbiota and T2DM has attracted much attention recently. Several researches have demonstrated that individuals with T2DM frequently exhibit a unique gut microbiota composition compared to healthy controls. For example, a study conducted on Indian individuals with T2DM identified alterations in eubacterial, archaeal, and eukaryotic components, highlighting pervasive dysbiosis in newly diagnosed and long-standing diabetic patients (29). Furthermore, metagenomic analyses have identified specific bacterial taxa associated with metabolic dysfunction in individuals with T2DM, such as the increased abundance of *Firmicutes* and diminished levels of butyrate-producing bacteria like the *Ruminococcaceae* (29). The animal experiment indicated significant alterations in gut microbial diversity and metabolite profiles, which were found to be closely associated with glucose metabolism and IR (30). Moreover, Mendelian randomization studies have further clarified the possible causality between gut microbiota and T2DM, identifying specific genera that may influence the risk of developing the disease (31, 32). These findings emphasize the potential for targeting gut microbiota as a treatment approach for managing T2DM and its associated complications.

DI-GM is a newly developed dietary index to capture dietary patterns that are beneficial or harmful to gut health. Our study preliminarily established a linear negative relationship between DI-GM and T2DM as well as IR. This provides some evidence that adhering to a dietary pattern promoting healthy gut microbiota identified by DI-GM may reduce the occurrence of T2DM and IR. The impact of diet on gut microbiota and its potential role in T2DM development is widely discussed. Recent researches have revealed that dietary patterns play important roles in affecting the gut microbiota composition and function, which can subsequently affect metabolic health and the development of conditions such as T2DM (33, 34). For instance, high-fiber diets were demonstrated to be related to improvements in gut microbiota diversity and the enrichment of beneficial bacteria, which can enhance glucose metabolism and reduce systemic inflammation in T2DM patients (35). Additionally, the Mediterranean diet, rich in polyphenols and healthy fats, has been shown to positively modulate gut microbiota composition, potentially lowering T2DM risk (36). Moreover, probiotic supplementation has been shown to significantly impact oxidative stress and inflammation biomarkers in diabetic patients (37). These findings suggested a promising new avenue for dietary interventions in managing T2DM.

Our findings revealed that BMI and inflammation markers (SII and SIRI) may mediated the relationship between DI-GM and T2DM. It is noteworthy that SII and SIRI mediated only approximately 2% of this association. However, we should not overlook this finding, as it provides theoretical support and direction for future longitudinal studies or interventional trials. Gut microbiota dysbiosis was reported to result in the excessive leakage of gram-negative bacterial products, such as lipopolysaccharides (LPS), which promote systemic low-grade inflammation and elevate the risk of metabolic disorders. This process leads to local endotoxemia in the small intestine and colon, particularly with an influx of gram-negative genera, including *Bacteroides*, *Prevotella*, and *Escherichia.* Such microbial changes represent a critical risk factor for obesity and IR, ultimately contributing to the development of T2DM (14, 38, 39). In addition, gut dysbiosis can lead to intestinal barrier disruption, permitting excessive leakage of LPS into the bloodstream. This triggers an inflammatory response, leading to IR and disrupted glucose homeostasis (14, 40, 41). These findings are consistent with our results. Our further analysis also revealed a significant mediating role of NEUT in the relationship between DI-GM and T2DM or IR. Previous studies also suggest that NEUT play an important role in the occurrence of IR, via secreted elastase (42). However, due to the cross-sectional study design, we need to interpret these findings with caution. Further studies are necessary to validate these findings and investigate the underlying mechanisms.

This study has several strengths. Firstly, DI-GM is a new novel dietary index, we are the first to investigate its correlation with T2DM and IR, as far as we know. Secondly, all data we used were from NHANES, a comprehensive nationally representative database, which utilizes a multistage sampling methodology to improve the reliability and robustness of the findings. Thirdly, potential confounders were adjusted to ensure consistent and reliable conclusions across various subgroups. Furthermore, multiple sensitivity analyses were conducted to evaluate the stability of the findings.

Several limitations exist in this study. Firstly, the cross-sectional design was unable to draw causal relationships. Secondly, although a variety of confounders were considered, unknown residual confounding factors cannot be excluded. Thirdly, dietary data were obtained through self-reported 24-h dietary recalls, which are prone to recall bias. However, potential errors were mitigated by averaging the results from two 24-h dietary recall interviews. Lastly, although our study did not include individuals under the age of 20, which would reduce the misclassification of T1DM, the possibility of residual inclusion of T1DM cases cannot be eliminated.

## 5 Conclusion

The newly proposed dietary index, i.e., DI-GM, was negatively correlated with the prevalence of T2DM, IR, and the risk markers of T2DM (FBG, FSI, HOMA-IR). In addition, we found the mediating role of BMI and inflammatory markers (SII and SIRI). Our results suggest that DI-GM is expected to identify the dietary patterns that are beneficial to gut health, thus, reducing the incidence of T2DM. Additional studies are required to verify and support our findings.

## Data Availability

The datasets presented in this study can be found in online repositories. The names of the repository/repositories and accession number(s) can be found at: https://wwwn.cdc.gov/nchs/nhanes/.
