# Supplementary material for: Linear association of the dietary index for gut microbiota with insulin resistance and type 2 diabetes mellitus in U.S. adults: the mediating role of body mass index and inflammatory markers
Source: Front Nutr. 2025 Mar 21;12:1557280. doi: 10.3389/fnut.2025.1557280 (PMC11968382; doi:10.3389/fnut.2025.1557280)
Supplement: Supplementary file 1 [file Table_1.docx]

Supplementary Material

# Supplementary Appendix

*Assessment of covariates*

**Race:** Race was classified as Non-Hispanic Black, Non-Hispanic White, Mexican American, and other (1).

**Education level:** Education level was classified as Less than High school, High school grad or equivalent, and College or above.

**Marital status:** Marital status was categorized as "Married or living with partner" or other.

**Poverty-to-income ratio (PIR):** The poverty-income ratio was derived from the ratio of self-reported family income to the appropriate poverty threshold and classified into three groups (<1.3, 1.3-3.5, >3.5) (2).

**Smoking status:** Smoking status was measured in the "smoking: cigarette use" questionnaire. In the “smoking: cigarette use” questionnaire, respondents were asked if s/he had smoked at least 100 cigarettes in their life, and smoked cigarettes when being questioned. If the respondent had smoked less than 100 cigarettes in their life, s/he was classified as a never smoker. If the respondent had smoked at least 100 cigarettes in his/her life and still smoked when s/he answered the questionnaire, s/he was classified as a current smoker. The respondent was classified as a former smoker if s/he had smoked at least 100 cigarettes in his/her life, and had quit smoking when s/he answered the questionnaire. Smoking status was categorized into never, former, and current smoker (3).

**Dinking status:** Drinking status was categorized as follows: never (consumed < 12 drinks in a lifetime), former (consumed ≥ 12 drinks in one year but not in the last year, or did not drink in the last year but consumed ≥ 12 drinks in a lifetime), mild (≤ 1 drink per day for females and ≤ 2 drinks per day for males), moderate (≤ 2 drinks per day for females and ≤ 3 drinks per day for males), or heavy (≥ 3 drinks per day for females and ≥ 4 drinks per day for males) (4).

**Physical activity (PA):** Physical activity was assessed based on questionnaires regarding work or recreational activity, with individuals engaging in vigorous or both moderate to vigorous levels considered active (Yes), else no (5).

**BMI**: BMI was defined as weight divided by height squared (kg/m^2^), and then categorized into <25, and ≥25 (Overweight or not).

**Hypertension:** Hypertension was diagnosed as the presence of any of the following: 1) Blood pressure measurements taken three or more times on different days all show that: systolic blood pressure (SBP) ≥ 140 mmHg or diastolic blood pressure (DBP) ≥ 90 mmHg; 2) self-reported diagnosis; 3) use of antihypertensive drugs (6).

**Hyperlipidemia:** Hyperlipidemia was diagnosed as the presence of any of the following: 1) TG ≥ 150mg/dL; 2) total cholesterol (TC) ≥ 200 mg/dL[5.18mmol/L]; 3) low-density lipoprotein (LDL) ≥ 130 mg/dL[3.37mmol/L]; 4) high-density lipoprotein (HDL) ≤ 40 mg/dL[1.04mmol/L] in males, ≤ 50 mg/dL[1.30mmol/L] in females; 5) Use of lipid-lowering drugs (6).

**Hyperuricemia:** Hyperuricemia was described as serum uric acid ≥ 416 μmol/L (7.0 mg/dL) in men or ≥ 357 μmol/L (6.0 mg/dL) in women (7).

**Cardiovascular disease (CVD):** Whether they had CVD was based on their health status in the questionnaire, which included " Ever told you had congestive heart failure," " Ever told you had angina/angina pectoris," "Ever told you that you had a heart attack," and "Ever told you that you had a stroke”. According to the answer "yes" or "no" to these questionnaires, we determine whether they have one or more of the above-questioned four clinical features of CVD (8).

# Supplementary Figures and Tables

**Supplementary Table S1. Components and scoring standards of DI-GM in NHANES.**

**Supplementary Table S2. The associations of DI-GM with T2DM and IR after multiple imputation.**

**Supplementary Table S3.** **The association between DI-GM and risk markers of T2DM after** **multiple imputation.**

**Supplementary Table S4. Unweighted multivariable logistic regression analysis of DI-GM with T2DM and IR.**

**Supplementary Table S5. Unweighted multivariable logistic regression analysis of DI-GM with T2DM risk makers.**

**Supplementary Table S6. The associations of DI-GM with T2DM and IR after further adjustment for liver and kidney function indicators and total energy intake.**

**Supplementary Table S7. The association between DI-GM and the risk factors of T2DM after further adjustment for liver and kidney function indicators and total energy intake.**

**Supplementary Table S8. The associations of DI-GM with T2DM and IR after excluding the participants with CVD (Participants = 9 452).**

**Supplementary Table S9. The association between DI-GM and the risk factors of T2DM after excluding the participants with CVD (Participants = 9 452).**

**Supplementary Table S10. The associations of DI-GM with T2DM and IR after excluding the participants with hyperuricemia (Participants = 8 306).**

**Supplementary Table S11. The association between DI-GM and the risk factors of T2DM after excluding the participants with hyperuricemia (Participants = 8 306).**

**Supplementary Figure S1 The mediation analysis of WBC, LYM and MONO on the association of DI-GM with T2DM and IR. The graphs in (A–C) represented the mediating role of WBC, LYM, and MONO, respectively.**

**Supplementary Table S1. Components and scoring standards of DI-GM in NHANES.**

| **Components of DI-GM** | **Food items included in NHANES** | **Scoring standards** |
| --- | --- | --- |
| **Beneficial to gut microbiota** | Avocados | For each component, a score of 1 if consumption at or above the sex-specific median, otherwise 0 |
|  | Broccoli |  |
|  | Chickpeas |  |
|  | Coffee |  |
|  | Cranberries |  |
|  | Fermented dairy (yogurt, cheese, kefir, sour cream, buttermilk) |  |
|  | Fiber |  |
|  | Green tea |  |
|  | Soybean (Soy milk, Tofu) |  |
|  | Whole grains |  |
| **Unfavorable to gut microbiota** | Refined grains | For each remaining component, a score of 0 if consumption at or above the sex-specific median, otherwise 1 |
|  | Processed meat |  |
|  | Red meat |  |
|  | High-fat diet (% energy) | 0 if consumption at or above 40% energy from fat, otherwise 1 |

Abbreviations: DI-GM, dietary index for gut microbiota; NHANES, National Health and Nutrition Examination Survey.

**Supplementary Table S2. The associations of DI-GM with T2DM and IR after multiple imputation.**

| **Outcomes** | **Model 1**  **OR (95%CI)** | ***P*-value** | **Model 2**  **OR (95%CI)** | ***P*-value** | **Model 3**  **OR (95%CI)** | ***P*-value** |
| --- | --- | --- | --- | --- | --- | --- |
| **T2DM** |  |  |  |  |  |  |
| DI-GM continuous | 0.93 (0.90, 0.96) | <0.001 | 0.89 (0.86, 0.93) | <0.001 | 0.93 (0.89, 0.97) | 0.001 |
| DI-GM group | |  |  |  |  |  |
| 0-3 | Reference |  | Reference |  | Reference |  |
| 4-5 | 0.82 (0.73, 0.92) | <0.001 | 0.78 (0.68, 0.88) | <0.001 | 0.85 (0.73, 1.00) | 0.005 |
| ≥6 | 0.72 (0.62, 0.84) | <0.001 | 0.60 (0.51, 0.72) | <0.001 | 0.73 (0.60, 0.88) | 0.001 |
| *P* for trend |  | <0.001 |  | <0.001 |  | 0.002 |
| **IR** |  |  |  |  |  |  |
| DI-GM continuous | 0.90 (0.88, 0.93) | <0.001 | 0.89 (0.86, 0.93) | <0.001 | 0.94 (0.90, 0.98) | 0.004 |
| DI-GM group | |  |  |  |  |  |
| 0-3 | Reference |  | Reference |  | Reference |  |
| 4-5 | 0.88 (0.76, 1.02) | 0.088 | 0.88 (0.76, 1.02) | 0.080 | 0.96 (0.81, 1.13) | 0.601 |
| ≥6 | 0.61 (0.51, 0.73) | <0.001 | 0.60 (0.50, 0.72) | <0.001 | 0.73 (0.60, 0.89) | 0.002 |
| *P* for trend |  | <0.001 |  | <0.001 |  | 0.002 |

Model 1: adjusted for no covariates;

Model 2: adjusted for age, gender, race;

Model 3: adjusted for all covariates (age, gender, race, education level, marital status, PIR, smoking status, drinking status, PA, BMI, hypertension, hyperlipidemia, hyperuricemia and CVD).

Abbreviations: OR, odds ratio; 95% CI, 95% confidence interval. T2DM, type 2 diabetes mellitus; IR, insulin resistance.

**Supplementary Table S3.** **The association between DI-GM and risk markers of T2DM after** **multiple imputation.**

| **Outcomes** | **Model 1**  **β (95% CI)** | ***P*-value** | **Model 2**  **β (95% CI)** | ***P*-value** | **Model 3**  **β (95% CI)** | ***P*-value** |
| --- | --- | --- | --- | --- | --- | --- |
| **FBG** |  |  |  |  |  |  |
| DI-GM continuous | -0.04 (-0.07, -0.02) | <0.001 | -0.06 (-0.08, -0.03) | <0.001 | -0.03 (-0.05, -0.00) | 0.031 |
| DI-GM group | |  |  |  |  |  |
| 0-3 | Reference |  | Reference |  | Reference |  |
| 4-5 | -0.17 (-0.25, -0.09) | <0.001 | -0.17 (-0.25, -0.10) | <0.001 | -0.13 (-0.20, -0.05) | 0.002 |
| ≥6 | -0.22 (-0.33, -0.12) | <0.001 | -0.27 (-0.37, -0.17) | <0.001 | -0.15 (-0.25, -0.05) | 0.003 |
| *P* for trend |  | <0.001 |  | <0.001 |  | 0.004 |
| **FSI** |  |  |  |  |  |  |
| DI-GM continuous | -0.61 (-0.75, -0.47) | <0.001 | -0.57 (-0.71, -0.44) | <0.001 | -0.26 (-0.39, -0.13) | <0.001 |
| DI-GM group | |  |  |  |  |  |
| 0-3 | Reference |  | Reference |  | Reference |  |
| 4-5 | -0.57 (-1.39, 0.25) | 0.171 | -0.55 (-1.37, 0.28) | 0.190 | -0.06 (-0.82, 0.71) | 0.880 |
| ≥6 | -2.53 (-3.25, -1.80) | <0.001 | -2.39 (-3.11, -1.67) | <0.001 | -1.10 (-1.79, -0.41) | 0.002 |
| *P* for trend |  | <0.001 |  | <0.001 |  | 0.001 |
| **HOMA-IR** | |  |  |  |  |  |
| DI-GM continuous | -0.22 (-0.27, -0.16) | <0.001 | -0.22 (-0.27, -0.16) | <0.001 | -0.11 (-0.16, -0.06) | <0.001 |
| DI-GM group | |  |  |  |  |  |
| 0-3 | Reference |  | Reference |  | Reference |  |
| 4-5 | -0.31 (-0.65, 0.03) | 0.071 | -0.31 (-0.65, 0.03) | 0.071 | -0.14 (-0.46, 0.18) | 0.379 |
| ≥6 | -0.94 (-1.24, -0.65) | <0.001 | -0.94 (-1.24, -0.65) | <0.001 | -0.51 (-0.80, -0.21) | <0.001 |
| *P* for trend |  | <0.001 |  | <0.001 |  | <0.001 |

Model 1: adjusted for no covariates;

Model 2: adjusted for age, gender, race;

Model 3: adjusted for all covariates (age, gender, race, education level, marital status, PIR, smoking status, drinking status, PA, BMI, hypertension, hyperlipidemia, hyperuricemia and CVD).

Abbreviations: FBG, fasting blood glucose; FSI, fasting serum insulin; HOMA-IR, homeostasis model assessment of insulin resistance.

**Supplementary Table S4. Unweighted multivariable logistic regression analysis of DI-GM with T2DM and IR.**

| **Outcomes** | **Model 1**  **OR (95%CI)** | ***P*-value** | **Model 2**  **OR (95%CI)** | ***P*-value** | **Model 3**  **OR (95%CI)** | ***P*-value** |
| --- | --- | --- | --- | --- | --- | --- |
| **T2DM** |  |  |  |  |  |  |
| DI-GM continuous | 0.92 (0.90, 0.95) | <0.001 | 0.88 (0.86, 0.91) | <0.001 | 0.93 (0.89, 0.98) | 0.005 |
| DI-GM group | |  |  |  |  |  |
| 0-3 | Reference |  | Reference |  | Reference |  |
| 4-5 | 0.83 (0.74, 0.93) | 0.002 | 0.78 (0.69, 0.88) | <0.001 | 0.85 (0.73, 1.00) | 0.047 |
| ≥6 | 0.72 (0.63, 0.82) | <0.001 | 0.60 (0.52, 0.69) | <0.001 | 0.74 (0.60, 0.91) | 0.006 |
| *P* for trend |  | <0.001 |  | <0.001 |  | 0.006 |
| **IR** |  |  |  |  |  |  |
| DI-GM continuous | 0.91 (0.88, 0.93) | <0.001 | 0.90 (0.88, 0.93) | <0.001 | 0.95 (0.91, 0.99) | 0.030 |
| DI-GM group | |  |  |  |  |  |
| 0-3 | Reference |  | Reference |  | Reference |  |
| 4-5 | 0.93 (0.83, 1.03) | 0.159 | 0.92 (0.82, 1.02) | 0.111 | 0.98 (0.82, 1.17) | 0.798 |
| ≥6 | 0.65 (0.58, 0.74) | <0.001 | 0.64 (0.56, 0.73) | <0.001 | 0.77 (0.62, 0.95) | 0.016 |
| *P* for trend |  | <0.001 |  | <0.001 |  | 0.014 |

Model 1: adjusted for no covariates;

Model 2: adjusted for age, gender, race;

Model 3: adjusted for all covariates (age, gender, race, education level, marital status, PIR, smoking status, drinking status, PA, BMI, hypertension, hyperlipidemia, hyperuricemia and CVD).

**Supplementary Table S5. Unweighted multivariable logistic regression analysis of DI-GM with T2DM risk makers.**

| **Outcomes** | **Model 1**  **β (95% CI)** | ***P*-value** | **Model 2**  **β (95% CI)** | ***P*-value** | **Model 3**  **β (95% CI)** | ***P*-value** |
| --- | --- | --- | --- | --- | --- | --- |
| **FBG** |  |  |  |  |  |  |
| DI-GM continuous | -0.07 (-0.09, -0.05) | <0.001 | -0.08 (-0.10, -0.06) | <0.001 | -0.05 (-0.07, -0.03) | <0.001 |
| DI-GM group | |  |  |  |  |  |
| 0-3 | Reference |  | Reference |  | Reference |  |
| 4-5 | -0.19 (-0.28, -0.10) | <0.001 | -0.20 (-0.29, -0.11) | <0.001 | -0.15 (-0.24, -0.07) | 0.001 |
| ≥6 | -0.30 (-0.40, -0.20) | <0.001 | -0.36 (-0.46, -0.26) | <0.001 | -0.23 (-0.33, -0.13) | <0.001 |
| *P* for trend |  | <0.001 |  | <0.001 |  | <0.001 |
| **FSI** |  |  |  |  |  |  |
| DI-GM continuous | -0.50 (-0.71, -0.30) | <0.001 | -0.49 (-0.70, -0.29) | <0.001 | -0.20 (-0.40, 0.01) | 0.057 |
| DI-GM group | |  |  |  |  |  |
| 0-3 | Reference |  | Reference |  | Reference |  |
| 4-5 | -0.43 (-1.25, 0.40) | 0.314 | -0.46 (-1.29, 0.36) | 0.272 | 0.00 (-0.80, 0.80) | 0.994 |
| ≥6 | -2.05 (-2.98, -1.13) | <0.001 | -2.02 (-2.95, -1.09) | <0.001 | -0.80 (-1.71,0.11) | 0.084 |
| *P* for trend |  | <0.001 |  | <0.001 |  | 0.077 |
| **HOMA-IR** | |  |  |  |  |  |
| DI-GM continuous | -0.21 (-0.30, -0.13) | <0.001 | -0.22 (-0.31, -0.14) | <0.001 | -0.12 (-0.20, -0.03) | 0.006 |
| DI-GM group | |  |  |  |  |  |
| 0-3 | Reference |  | Reference |  | Reference |  |
| 4-5 | -0.30 (-0.63, 0.04) | 0.08 | -0.32 (-0.65, 0.01) | 0.059 | -0.15 (-0.48, 0.17) | 0.358 |
| ≥6 | -0.91 (-1.29, -0.54) | <0.001 | -0.96 (-1.33, -0.58) | <0.001 | -0.51 (-0.88, -0.14) | 0.007 |
| *P* for trend |  | <0.001 |  | <0.001 |  | 0.006 |

Model 1: adjusted for no covariates;

Model 2: adjusted for age, gender, race;

Model 3: adjusted for all covariates (age, gender, race, education level, marital status, PIR, smoking status, drinking status, PA, BMI, hypertension, hyperlipidemia, hyperuricemia and CVD).

**Supplementary Table S6. The associations of DI-GM with T2DM and IR after further adjustment for liver and kidney function indicators and total energy intake.**

| **Outcomes** | **Model 4**  **OR (95%CI)** | ***P*-value** | **Model 5**  **OR (95%CI)** | ***P*-value** |
| --- | --- | --- | --- | --- |
| **T2DM** |  |  |  |  |
| DI-GM continuous | 0.94 (0.90, 0.99) | 0.012 | 0.94 (0.89, 0.98) | 0.005 |
| DI-GM group | |  |  |  |
| 0-3 | Reference |  | Reference |  |
| 4-5 | 0.85 (0.73, 1.00) | 0.051 | 0.85 (0.73, 1.00) | 0.047 |
| ≥6 | 0.76 (0.62, 0.94) | 0.013 | 0.74 (0.60, 0.92) | 0.006 |
| *P* for trend |  | 0.014 |  | 0.007 |
| **IR** |  |  |  |  |
| DI-GM continuous | 0.95 (0.91, 0.99) | 0.024 | 0.95 (0.90, 0.99) | 0.025 |
| DI-GM group | |  |  |  |
| 0-3 | Reference |  | Reference |  |
| 4-5 | 0.97 (0.81, 1.16) | 0.720 | 0.98 (0.82, 1.17) | 0.823 |
| ≥6 | 0.76 (0.62, 0.94) | 0.012 | 0.76 (0.61, 0.95) | 0.015 |
| *P* for trend |  | 0.011 |  | 0.013 |

Model 3: adjusted for all covariates (age, gender, race, education level, marital status, PIR, smoking status, drinking status, PA, BMI, hypertension, hyperlipidemia, hyperuricemia and CVD).

Model 4: Model 3 + AST, ALT, GGT, ALP, SCR, BUN

Model 5: Model 3 + total energy intake (kcal/day)

Abbreviations: AST, aspartate aminotransferase; ALT, alanine aminotransferase; GGT, γ-glutamyl transpeptidase; ALP, alkaline phosphatase; SCR, serum creatinine; BUN, blood urea nitrogen.

**Supplementary Table S7. The association between DI-GM and the risk factors of T2DM after further adjustment for liver and kidney function indicators and total energy intake.**

| **Outcomes** | **Model 4**  **OR (95%CI)** | ***P*-value** | **Model 5**  **OR (95%CI)** | ***P*-value** |
| --- | --- | --- | --- | --- |
| **FBG** |  |  |  |  |
| DI-GM continuous | -0.03 (-0.05, -0.00) | 0.044 | -0.04 (-0.06, -0.01) | 0.005 |
| DI-GM group | |  |  |  |
| 0-3 | Reference |  | Reference |  |
| 4-5 | -0.13 (-0.21, -0.04) | 0.005 | -0.14 (-0.23, -0.06) | 0.002 |
| ≥6 | -0.15 (-0.25, -0.05) | 0.005 | -0.19 (-0.30, -0.09) | <0.001 |
| *P* for trend |  | 0.007 |  | <0.001 |
| **FSI** |  |  |  |  |
| DI-GM continuous | -0.28 (-0.42, -0.13) | <0.001 | -0.33 (-0.48, -0.17) | <0.001 |
| DI-GM group | |  |  |  |
| 0-3 | Reference |  | Reference |  |
| 4-5 | -0.17 (-1.06, 0.72) | 0.699 | -0.18 (-1.08, 0.72) | 0.688 |
| ≥6 | -1.24 (-2.05, -0.44) | 0.003 | -1.42 (-2.27, -0.57) | 0.001 |
| *P* for trend |  | 0.002 |  | <0.001 |
| **HOMA-IR** | |  |  |  |
| DI-GM continuous | -0.11 (-0.17, -0.05) | <0.001 | -0.14 (-0.20, -0.08) | <0.001 |
| DI-GM group | |  |  |  |
| 0-3 | Reference |  | Reference |  |
| 4-5 | -0.19 (-0.56, 0.18) | 0.310 | -0.21 (-0.58, 0.16) | 0.263 |
| ≥6 | -0.55 (-0.89, -0.22) | 0.002 | -0.65 (-1.00, -0.30) | <0.001 |
| *P* for trend |  | <0.001 |  | <0.001 |

Model 3: adjusted for all covariates (age, gender, race, education level, marital status, PIR, smoking status, drinking status, PA, BMI, hypertension, hyperlipidemia, hyperuricemia and CVD).

Model 4: Model 3 + AST, ALT, GGT, ALP, SCR, BUN

Model 5: Model 3 + total energy intake (kcal/day)

Abbreviations: AST, aspartate aminotransferase; ALT, alanine aminotransferase; GGT, γ-glutamyl transpeptidase; ALP, alkaline phosphatase; SCR, serum creatinine; BUN, blood urea nitrogen.

**Supplementary Table S8. The associations of DI-GM with T2DM and IR after excluding the participants with CVD (Participants = 9 452).**

| **Outcomes** | **Model 1**  **OR (95%CI)** | ***P*-value** | **Model 2**  **OR (95%CI)** | ***P*-value** | **Model 3**  **OR (95%CI)** | ***P*-value** |
| --- | --- | --- | --- | --- | --- | --- |
| **T2DM** |  |  |  |  |  |  |
| DI-GM continuous | 0.93 (0.89, 0.98) | 0.002 | 0.89 (0.85, 0.93) | <0.001 | 0.93 (0.88, 0.98) | 0.012 |
| DI-GM group | |  |  |  |  |  |
| 0-3 | Reference |  | Reference |  | Reference |  |
| 4-5 | 0.85 (0.72, 0.99) | 0.043 | 0.80 (0.67, 0.94) | 0.009 | 0.85 (0.71, 1.01) | 0.060 |
| ≥6 | 0.75 (0.63, 0.90) | 0.002 | 0.61 (0.49, 0.75) | <0.001 | 0.74 (0.59, 0.93) | 0.010 |
| *P* for trend |  | 0.003 |  | <0.001 |  | 0.011 |
| **IR** |  |  |  |  |  |  |
| DI-GM continuous | 0.90 (0.87, 0.94) | <0.001 | 0.90 (0.87, 0.94) | <0.001 | 0.95 (0.91, 1.00) | 0.047 |
| DI-GM group | |  |  |  |  |  |
| 0-3 | Reference |  | Reference |  | Reference |  |
| 4-5 | 0.93 (0.79, 1.10) | 0.404 | 0.92 (0.78, 1.09) | 0.355 | 1.01 (0.83, 1.23) | 0.907 |
| ≥6 | 0.64 (0.52, 0.78) | <0.001 | 0.63 (0.51, 0.77) | <0.001 | 0.77 (0.62, 0.97) | 0.030 |
| *P* for trend |  | <0.001 |  | <0.001 |  | 0.027 |

Model 1: adjusted for no covariates;

Model 2: adjusted for age, gender, race;

Model 3: adjusted for all covariates (age, gender, race, education level, marital status, PIR, smoking status, drinking status, PA, BMI, hypertension, hyperlipidemia, and hyperuricemia).

**Supplementary Table S9. The association between DI-GM and the risk factors of T2DM after excluding the participants with CVD (Participants = 9 452).**

| **Outcomes** | **Model 1**  **β (95% CI)** | ***P*-value** | **Model 2**  **β (95% CI)** | ***P*-value** | **Model 3**  **β (95% CI)** | ***P*-value** |
| --- | --- | --- | --- | --- | --- | --- |
| **FBG** |  |  |  |  |  |  |
| DI-GM continuous | -0.04 (-0.06, -0.01) | 0.004 | -0.05 (-0.08, -0.03) | <0.001 | -0.02 (-0.05, 0.00) | 0.064 |
| DI-GM group | |  |  |  |  |  |
| 0-3 | Reference |  | Reference |  | Reference |  |
| 4-5 | -0.14 (-0.24, -0.04) | 0.005 | -0.15 (-0.24, -0.06) | 0.002 | -0.11 (-0.20, -0.02) | 0.016 |
| ≥6 | -0.20 (-0.30, -0.09) | <0.001 | -0.25 (-0.35, -0.15) | <0.001 | -0.15 (-0.25, -0.05) | 0.006 |
| *P* for trend |  | <0.001 |  | <0.001 |  | 0.007 |
| **FSI** |  |  |  |  |  |  |
| DI-GM continuous | -0.56 (-0.72, -0.41) | <0.001 | -0.52 (-0.68, -0.36) | <0.001 | -0.19 (-0.34, -0.05) | 0.011 |
| DI-GM group | |  |  |  |  |  |
| 0-3 | Reference |  | Reference |  | Reference |  |
| 4-5 | -0.41 (-1.42, 0.59) | 0.414 | -0.40 (-1.42, 0.61) | 0.430 | 0.06 (-0.87, 0.99) | 0.895 |
| ≥6 | -2.23 (-3.09, -1.37) | <0.001 | -2.08 (-2.94, -1.22) | <0.001 | -0.77 (-1.59, 0.05) | 0.065 |
| *P* for trend |  | <0.001 |  | <0.001 |  | 0.044 |
| **HOMA-IR** | |  |  |  |  |  |
| DI-GM continuous | -0.21 (-0.27, -0.14) | <0.001 | -0.20 (-0.27, -0.14) | <0.001 | -0.10 (-0.16, -0.03) | 0.003 |
| DI-GM group | |  |  |  |  |  |
| 0-3 | Reference |  | Reference |  | Reference |  |
| 4-5 | -0.27 (-0.70, 0.16) | 0.212 | -0.28 (-0.71, 0.15) | 0.204 | -0.12 (-0.53, 0.28) | 0.547 |
| ≥6 | -0.86 (-1.22, -0.50) | <0.001 | -0.86 (-1.22, -0.49) | <0.001 | -0.43 (-0.79, -0.07) | 0.019 |
| *P* for trend |  | <0.001 |  | <0.001 |  | 0.013 |

Model 1: adjusted for no covariates;

Model 2: adjusted for age, gender, race;

Model 3: adjusted for all covariates (age, gender, race, education level, marital status, PIR, smoking status, drinking status, PA, BMI, hypertension, hyperlipidemia, and hyperuricemia).

**Supplementary Table S10. The associations of DI-GM with T2DM and IR after excluding the participants with hyperuricemia (Participants = 8 306).**

| **Outcomes** | **Model 1**  **OR (95%CI)** | ***P-*value** | **Model 2**  **OR (95%CI)** | ***P*-value** | **Model 3**  **OR (95%CI)** | ***P*-value** |
| --- | --- | --- | --- | --- | --- | --- |
| **T2DM** |  |  |  |  |  |  |
| DI-GM continuous | 0.92 (0.88, 0.97) | <0.001 | 0.87 (0.83, 0.92) | <0.001 | 0.92 (0.87, 0.98) | 0.007 |
| DI-GM group | |  |  |  |  |  |
| 0-3 | Reference |  | Reference |  | Reference |  |
| 4-5 | 0.81 (0.68, 0.97) | 0.021 | 0.76 (0.63, 0.91) | 0.004 | 0.82 (0.67, 0.99) | 0.038 |
| ≥6 | 0.68 (0.56, 0.84) | <0.001 | 0.55 (0.44, 0.69) | <0.001 | 0.69 (0.54, 0.89) | 0.004 |
| *P* for trend |  | <0.001 |  | <0.001 |  | 0.004 |
| **IR** |  |  |  |  |  |  |
| DI-GM continuous | 0.88 (0.84, 0.93) | <0.001 | 0.88 (0.83, 0.93) | <0.001 | 0.92 (0.87, 0.98) | 0.007 |
| DI-GM group | |  |  |  |  |  |
| 0-3 | Reference |  | Reference |  | Reference |  |
| 4-5 | 0.83 (0.69, 1.00) | 0.049 | 0.82 (0.69, 0.99) | 0.037 | 0.89 (0.73, 1.07) | 0.212 |
| ≥6 | 0.54 (0.43, 0.68) | <0.001 | 0.53 (0.42, 0.66) | <0.001 | 0.64 (0.50, 0.82) | <0.001 |
| *P* for trend |  | <0.001 |  | <0.001 |  | <0.001 |

Model 1: adjusted for no covariates;

Model 2: adjusted for age, gender, race;

Model 3: adjusted for all covariates (age, gender, race, education level, marital status, PIR, smoking status, drinking status, PA, BMI, hypertension, hyperlipidemia, and CVD).

**Supplementary Table S11. The association between DI-GM and the risk factors of T2DM after excluding the participants with hyperuricemia (Participants = 8 306).**

| **Outcomes** | **Model 1**  **β (95% CI)** | ***P-*value** | **Model 2**  **β (95% CI)** | ***P*-value** | **Model 3**  **β (95% CI)** | ***P*-value** |
| --- | --- | --- | --- | --- | --- | --- |
| **FBG** |  |  |  |  |  |  |
| DI-GM continuous | -0.05 (-0.08, -0.02) | 0.001 | -0.06 (-0.09, -0.04) | <0.001 | -0.03 (-0.06, -0.00) | 0.024 |
| DI-GM group | |  |  |  |  |  |
| 0-3 | Reference |  | Reference |  | Reference |  |
| 4-5 | -0.22 (-0.33, -0.12) | <0.001 | -0.24 (-0.33, -0.14) | <0.001 | -0.19 (-0.29, -0.09) | <0.001 |
| ≥6 | -0.26 (-0.38, -0.14) | <0.001 | -0.32 (-0.43, -0.21) | <0.001 | -0.20 (-0.32, -0.09) | <0.001 |
| *P* for trend |  | <0.001 |  | <0.001 |  | 0.001 |
| **FSI** |  |  |  |  |  |  |
| DI-GM continuous | -0.49 (-0.67, -0.31) | <0.001 | -0.47 (-0.64, -0.29) | <0.001 | -0.17 (-0.32, -0.01) | 0.035 |
| DI-GM group | |  |  |  |  |  |
| 0-3 | Reference |  | Reference |  | Reference |  |
| 4-5 | -0.52 (-1.47, 0.42) | 0.275 | -0.54 (-1.49, 0.41) | 0.262 | -0.11 (-0.95, 0.72) | 0.792 |
| ≥6 | -2.12 (-2.98, -1.27) | <0.001 | -2.05 (-2.89, -1.21) | <0.001 | -0.85 (-1.62, -0.07) | 0.032 |
| P for trend |  | <0.001 |  | <0.001 |  | 0.023 |
| **HOMA-IR** | |  |  |  |  |  |
| DI-GM continuous | -0.19 (-0.26, -0.13) | <0.001 | -0.20 (-0.26, -0.13) | <0.001 | -0.09 (-0.15, -0.04) | 0.002 |
| DI-GM group | |  |  |  |  |  |
| 0-3 | Reference |  | Reference |  | Reference |  |
| 4-5 | -0.35 (-0.71, 0.01) | 0.057 | -0.37 (-0.73, -0.01) | 0.047 | -0.22 (-0.56, 0.11) | 0.193 |
| ≥6 | -0.88 (-1.19, -0.56) | <0.001 | -0.90 (-1.21, -0.59) | <0.001 | -0.49 (-0.79, -0.20) | 0.001 |
| *P* for trend |  | <0.001 |  | <0.001 |  | <0.001 |

Model 1: adjusted for no covariates;

Model 2: adjusted for age, gender, race;

Model 3: adjusted for all covariates (age, gender, race, education level, marital status, PIR, smoking status, drinking status, PA, BMI, hypertension, hyperlipidemia, and CVD).


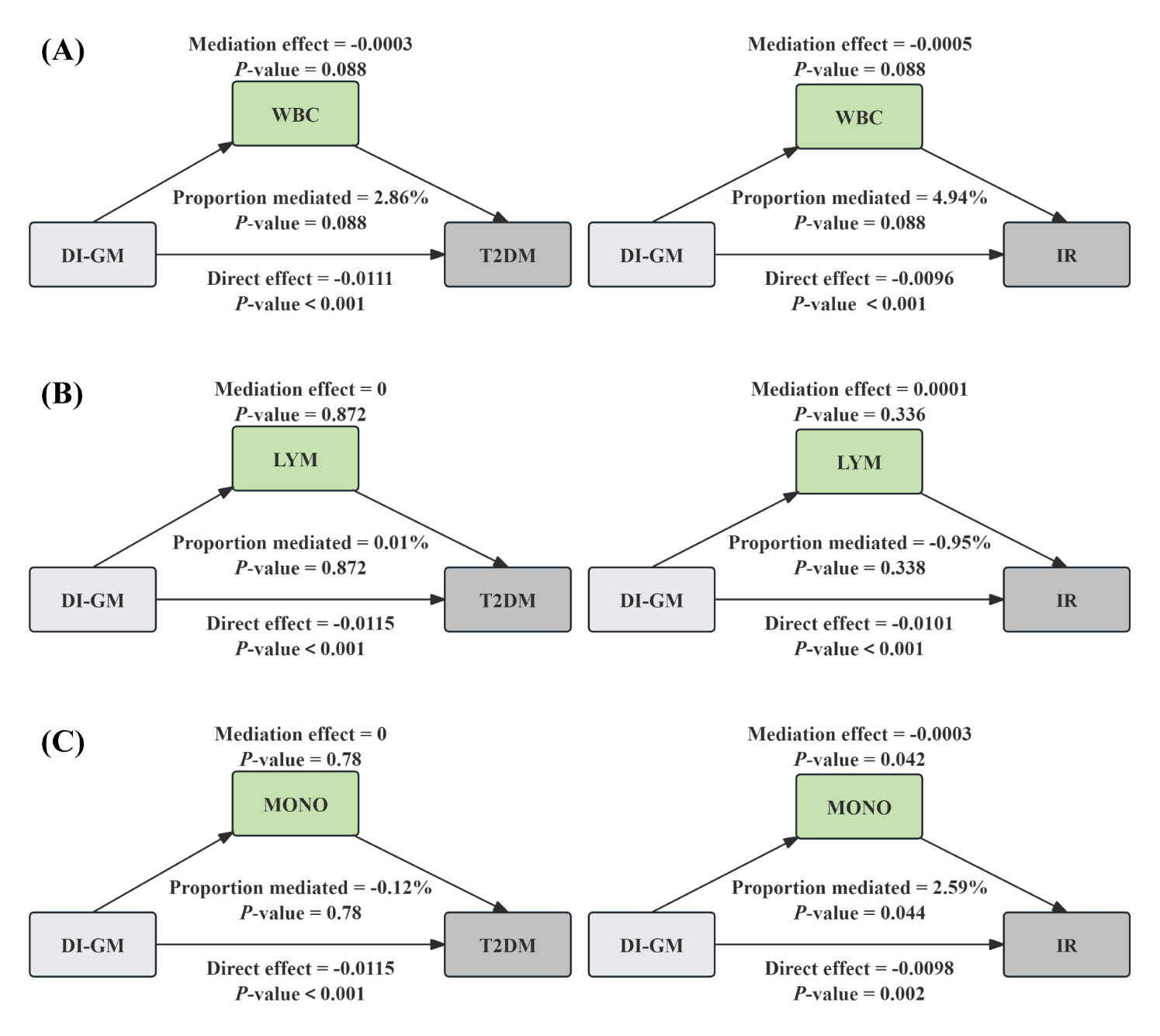


Supplementary Figure S1 The mediation analysis of WBC, LYM, and MONO on the association of DI-GM with T2DM and IR. The graphs in (A–C) represented the mediating role of WBC, LYM, and MONO, respectively.

**REFERENCES**

1. Tsai J, Homa DM, Gentzke AS, Mahoney M, Sharapova SR, Sosnoff CS, et al. Exposure to Secondhand Smoke Among Nonsmokers - United States, 1988-2014. *MMWR Morb Mortal Wkly Rep* (2018) **67**: 1342-46. doi:10.15585/mmwr.mm6748a3

2. Yang H, Liu Y, Huang Z, Deng G. Sex-specific associations of serum cotinine levels with depressive symptoms and sleep disorders in American adults: NHANES 2007-2014. *Front Psychiatry* (2024) **15**: 1434116. doi:10.3389/fpsyt.2024.1434116

3. Wang K, Zhao Y, Nie J, Xu H, Yu C, Wang S. Higher HEI-2015 Score Is Associated with Reduced Risk of Depression: Result from NHANES 2005-2016. *Nutrients* (2021) **13**. doi:10.3390/nu13020348

4. Zhang X, Yang Q, Huang J, Lin H, Luo N, Tang H. Association of the newly proposed dietary index for gut microbiota and depression: the mediation effect of phenotypic age and body mass index. *Eur Arch Psychiatry Clin Neurosci* (2024). doi:10.1007/s00406-024-01912-x

5. Liu AB, Lin YX, Meng TT, Tian P, Chen JL, Zhang XH, et al. Associations of the cardiometabolic index with insulin resistance, prediabetes, and diabetes in U.S. adults: a cross-sectional study. *BMC Endocr Disord* (2024) **24**: 217. doi:10.1186/s12902-024-01676-4

6. Yan Y, Li J. Association of dietary anthocyanidins intake with all-cause mortality and cardiovascular diseases mortality in USA adults: a prospective cohort study. *Sci Rep* (2024) **14**: 26595. doi:10.1038/s41598-024-76805-z

7. Ma Y, Hu Q, Yang D, Zhao Y, Bai J, Mubarik S, et al. Combined exposure to multiple metals on serum uric acid in NHANES under three statistical models. *Chemosphere* (2022) **301**: 134416. doi:10.1016/j.chemosphere.2022.134416

8. Qu S, Zhang Z, Ju R, Li Z, Leng J, Xue Z, et al. Association between the female hormone intake and cardiovascular disease in the women: a study based on NHANES 1999-2020. *BMC Public Health* (2024) **24**: 3578. doi:10.1186/s12889-024-21001-x
